# Impact of Intermittent Apnea on Myocardial Tissue Oxygenation—A Study Using Oxygenation-Sensitive Cardiovascular Magnetic Resonance

**DOI:** 10.1371/journal.pone.0053282

**Published:** 2013-01-03

**Authors:** Dominik P. Guensch, Kady Fischer, Jacqueline A. Flewitt, Matthias G. Friedrich

**Affiliations:** 1 Stephenson Cardiovascular MR Centre at the Libin Cardiovascular Institute of Alberta, Departments of Cardiac Sciences and Radiology, University of Calgary, Calgary, Alberta, Canada; 2 CMR Research Centre at the Montreal Heart Institute, Université de Montréal, Montreal, Quebec, Canada; University Hospital of Würzburg, Germany

## Abstract

**Background:**

Carbon dioxide (CO_2_) is a recognized vasodilator of myocardial blood vessels that leads to changes in myocardial oxygenation through the recruitment of the coronary flow reserve. Yet, it is unknown whether changes of carbon dioxide induced by breathing maneuvers can be used to modify coronary blood flow and thus myocardial oxygenation. Oxygenation-sensitive cardiovascular magnetic resonance (CMR) using the blood oxygen level-dependent (BOLD) effect allows for non-invasive monitoring of changes of myocardial tissue oxygenation. We hypothesized that mild hypercapnia induced by long breath-holds leads to changes in myocardial oxygenation that can be detected by oxygenation-sensitive CMR.

**Methods and Results:**

In nine anaesthetized and ventilated pigs, 60s breath-holds were induced. Left ventricular myocardial and blood pool oxygenation changes, as monitored by oxygenation-sensitive CMR using a T2*-weighted steady-state-free-precession (SSFP) sequence at 1.5T, were compared to changes of blood gas levels obtained immediately prior to and after the breath-hold. Long breath-holds resulted in an increase of paCO_2_, accompanied by a decrease of paO_2_ and pH. There was a significant decrease of blood pressure, while heart rate did not change. A decrease in the left ventricular blood pool oxygenation was observed, which was similar to drop in SaO_2_. Oxygenation in the myocardial tissue however, was maintained throughout the period. Changes in myocardial oxygenation were strongly correlated with the change in paCO_2_ during the breath-hold (r = 0.90, *p* = 0.010).

**Conclusion:**

Despite a drop in blood oxygen levels, myocardial oxygenation is maintained throughout long breath-holds and is linearly correlated with the parallel increase of arterial CO_2_, a known coronary vasodilator. Breathing maneuvers in combination with oxygenation-sensitive CMR may be useful as a diagnostic test for coronary artery function.

## Introduction

Carbon dioxide (CO_2_) is a potent vasodilator in the cerebrovascular system [1], [2]. With perturbations as little as such caused by breath holding can induce changes in cerebral blood flow. Recent data from our group indicate that this is paralleled by an increase in myocardial blood flow [3], [4]. However, there is little information on the utility of CO_2_ as a vasodilator for diagnostic testing.

Oxygenation-sensitive CMR detects changes of haemoglobin oxygenation by making use of the fact that its magnetic properties change when transitioning from oxygenated to deoxygenated status: While oxygenated haemoglobin (oxyHb) is diamagnetic exhibiting a weak stabilization of the magnetic field surrounding the molecule, de-oxygenated haemoglobin (de-oxyHb) is paramagnetic, de-stabilizing the surrounding field and thereby leading to a loss of magnetic field homogeneity. T2* weighted CMR protocols sensitive to this “blood oxygen level-dependent (BOLD) effect” may show a regional signal intensity (SI) drop of tissue with such a relative increase of de-oxyHb [5], [6] or a shortening in T2* time, as seen in myocardial ischemia [7]. Vice versa, increasing blood flow without a matching increase of oxygen consumption leads to a decrease in de-oxyHb and thus to an increased SI. Ogawa et al. used this to detect small variations of regional blood flow due to activation of brain areas in functional magnetic resonance imaging (fMRI) of the brain [8]. Using adenosine-induced coronary vasodilation, we could recently show that the vasodilatory effect leads to a measurable SI increase, which was linearly related to coronary sinus blood oxygenation yet not to blood flow [9].

We hypothesized that long-breath-hold induced hypercapnia leads to changes of myocardial haemoglobin oxygenation, which can be detected by oxygenation-sensitive CMR.

## Materials and Methods

### Experimental Protocol

Nine juvenile male pigs (24.3±0.2 kg) were pre-medicated with 600 mg Ketamine, 10 mg Midazolam and 2 mg Fentanyl i.m., then anaesthetized with 20–25 mg/kg Thiopental to establish an appropriate anaesthesia depth. They were intubated with a standard cuffed endotracheal tube (ID 5.5–6 mm) and ventilated with a Harvard Ventilator. Anaesthesia was maintained with an intravenous drip (1–3 mg/h Midazolam, 1.6–4.8 mg/h Fentanyl) and a nitrous oxide/Isoflurane (0.6–1.5%) gas narcosis. To prevent arrhythmia, the animals received a continuous Lidocaine infusion (1 mg/min). The right carotid artery and the femoral artery were cannulated for invasive blood pressure and arterial blood gas measurements throughout the experiment. The left jugular and femoral vein were cannulated for intravenous infusions. Monitoring of anaesthesia and haemodynamics included EtCO_2_, FiO_2_/FiN_2_O, 3-lead ECG, invasive blood pressure and arterial blood gases. After preparation, the animals were transferred to a clinical 1.5T MRI system (Avanto®, Siemens Healthcare, Erlangen, Germany). Custom 12 m long ventilator tubing connected the ventilator from outside the MR suite. Blood gases were adjusted to a target paO_2_ of 100 mmHg and a paCO_2_ of 40 mmHg. Then, BOLD-sensitive steady-state-free-precession (SSFP) cine images were acquired in mid left-ventricular short axis views (slice thickness 10 mm, TE 2.78 ms, TR 5.56 ms, flip angle 90°, FOV 280×157.5, matrix 128×72) [9], [10]. Each cine was composed of 20 phases covering the entire cardiac cycle, obtained by retrospective ECG gating. BOLD-SSFP cines were acquired during a 1 min breath-hold. Immediately after resuming ventilation an arterial blood sample was taken to determine the changes in blood gas levels over the one minute breath-hold. Blood gases were utilized to calculate the approximate arterial haemoglobin saturation using a dissociation curve tool [11] based on the equations of Kelman and Severinghaus [12], [13].

### Image Analysis

**Figure 1:**
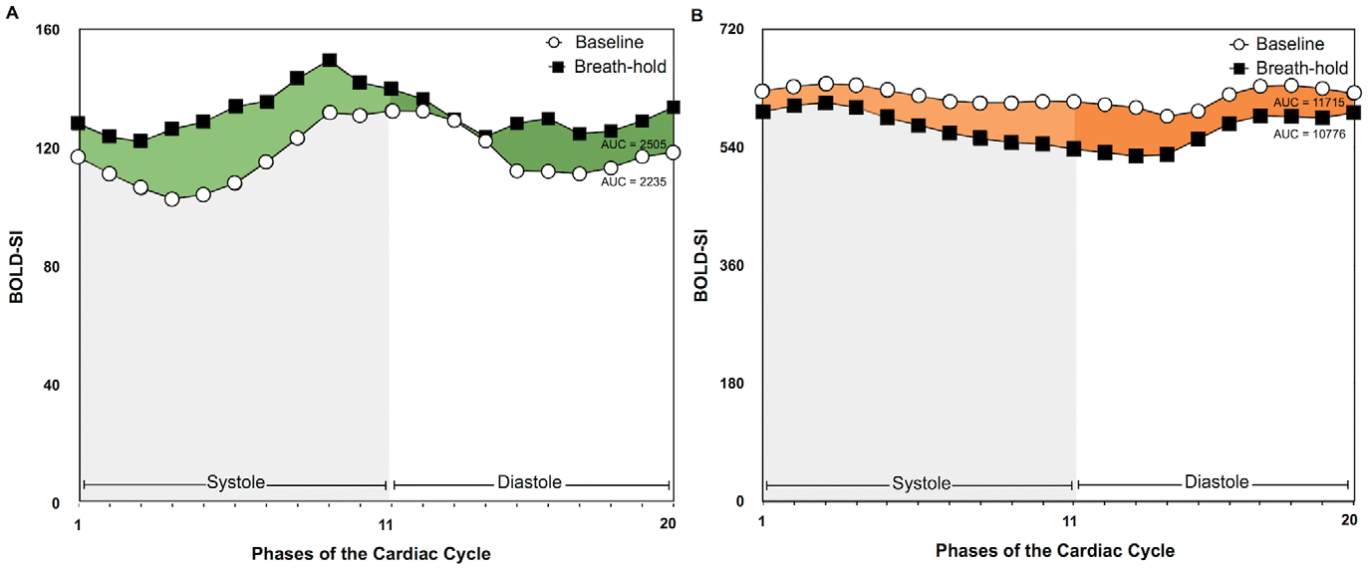
Myocardial and blood pool SI throughout the cardiac cycle. The area under the curve was calculated from the absolute BOLD-SI values of the 20 phases of the cardiac cycle from images obtained at the start and at the end of a breath-hold of both the myocardium (**A**) and blood pool (**B**) of one subject.

The images were analyzed with certified software for CMR image analysis (cmr^42^, Circle Cardiovascular Imaging Inc., Calgary, AB, Canada). Image quality was graded prior to SI measurement using visual assessment based on a 1–4 scale: 1 = good image quality, 2 = mildly impaired image quality resulting in <10% of the total myocardial area excluded, 3 = limited image quality resulting in >10% of the myocardium excluded, 4 = a severely non-analyzable image. The mean myocardial SI in the BOLD-sensitive images was automatically calculated after manual tracing of endocardial and epicardial contours in all images of each cine series. Additionally, a region of interest was defined in the centre of the left ventricular (LV) lumen for assessing SI changes in the arterial blood during the breath-hold. For the entire cardiac cycle, the area under the curve (AUC) was calculated from the signal intensity of all 20 phases (Figure 1) and expressed as percent change SI between baseline and the end of the breath-hold.

### Statistical Analysis

To determine the SI changes resulting from apnea, the AUC of the first two image sequences were compared to those of the last two images of the breath-hold using a paired t-test. Differences in blood gases and cardiovascular parameters over the breath-hold were analyzed with a paired t-test as well. Pearson’s correlation was performed to determine if there were relationships between the %-change SI and changes in blood gases, heart rate and blood pressure. Values are expressed as mean±SEM. Statistical analysis was calculated with GraphPad Prism (GraphPad Software, San Diego, CA) and deemed significant if *p*<0.05.

### Ethics Statement

This study was conducted in accordance with the “*Guide to the Care and Use of Experimental Animals”* by the Canadian Council on Animal Care. It was approved by the local “*Animal Care and Use Board*” and the institutional ethics committee.

## Results

One pig was excluded due to a pre-existing severe myocardial wall motion abnormality at baseline. Two pigs died in a sudden cardiac arrest during the preparation of the blood vessels, leaving 6 pigs for the data analysis. Overall, BOLD image quality was good in this study as visual assessment yielded a mean score of 1.3±0.3. One pig had 10%–15% of the myocardium excluded in the anterolateral and inferolateral segments due to susceptibility artifacts and two other pigs had <10% exclusions in the inferoseptal, inferior and inferolateral segments.

### Blood Gases and Cardiovascular Parameters

The paCO_2_ significantly increased from 41±0.4 to 47±1 mmHg during apnea (*p*<0.001) accompanied by a significant decrease in pH from 7.40±0.02 to 7.35±0.01 (*p* = 0.009) as shown in Table 1. Also, there was a significant decrease in paO_2_ from 100±2 to 65±5 mmHg (*p* = 0.003). As a result of the changing paO_2_, paCO_2_ and pH levels, the calculated SaO_2_ dropped by 9.9±3.5% (*p* = 0.037). There was no change in heart rate but all animals, however, showed a significant drop in blood pressures (*p*<0.05).

**Table 1:** Blood gas, blood pressure and heart rate analysis.

	Baseline	After Apnea	*p* Value
	(n = 6)	(n = 6)	
**Arterial blood gases [mmHg]**			
paCO_2_	41±0.4	47±1	<0.001
paO_2_	100±2	65±5	0.003
pH	7.40±0.02	7.35±0.01	0.009
**Arterial blood pressures [mmHg]**			
Systolic	100±11	85±18	0.013
Mean	74±9	64±13	0.035
Diastolic	59±9	50±11	0.029
**Heart rate [beats/min]**	110±14	114±14	0.448

Mean (±SEM) arterial partial pressures (mmHg) of blood gases, invasive blood pressures from the femoral artery and heart rate (n = 6).

### CMR Results

**Figure 2:**
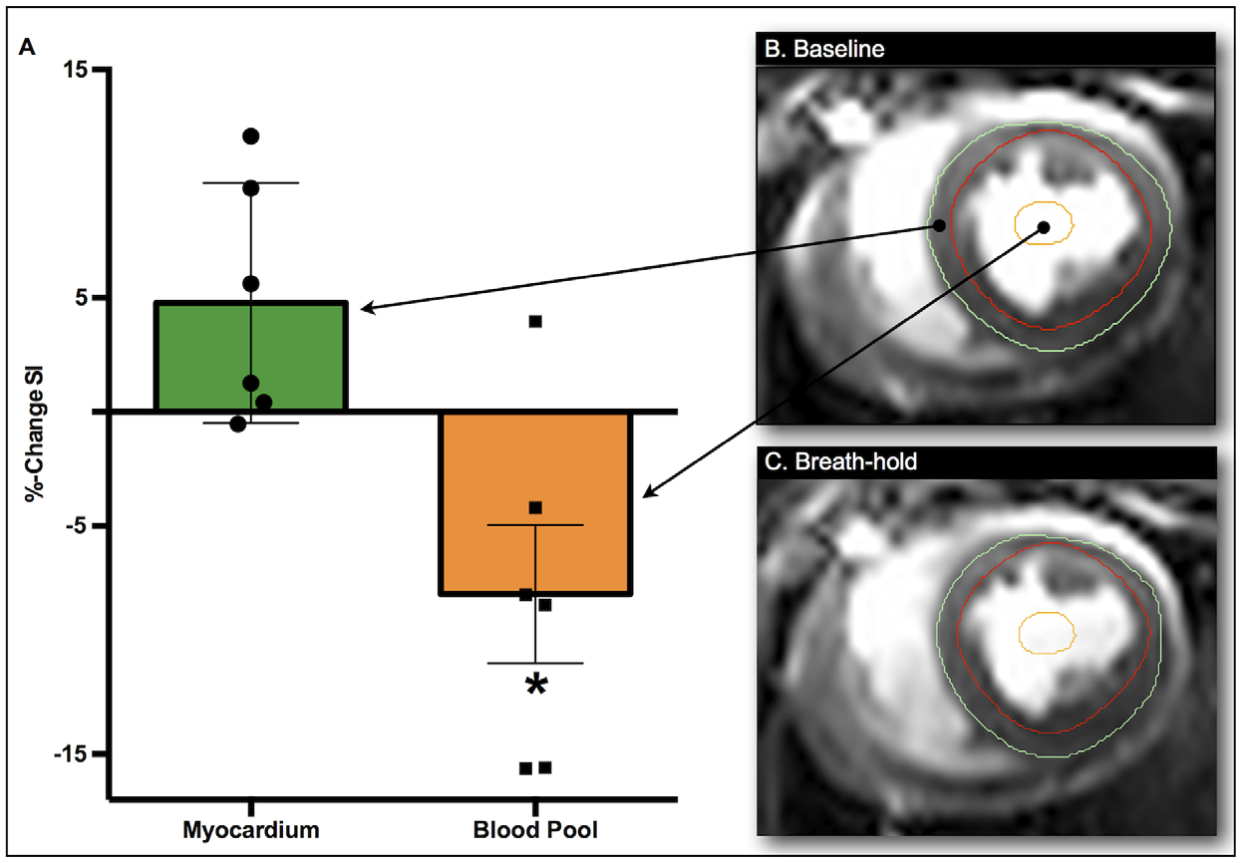
Change in arterial blood pool and myocardial tissue oxygenation during a 60 seconds breath-hold. Phase 11 of the cardiac cycle representing end-systole is presented for both the image from baseline (**B**) and the end of the breath-hold (**C**). The analyzed myocardial region is outlined on the BOLD-CMR image by the endocardial (red) and epicardial (green) contours resulting in a trend toward a mean SI increase (+4.8%; *p* = 0.077, n = 6) (**A**). The orange contour depicts a region of interest in the blood pool, with a significant mean SI decrease (-8.0%; *p* = 0.047).

**Figure 3:**
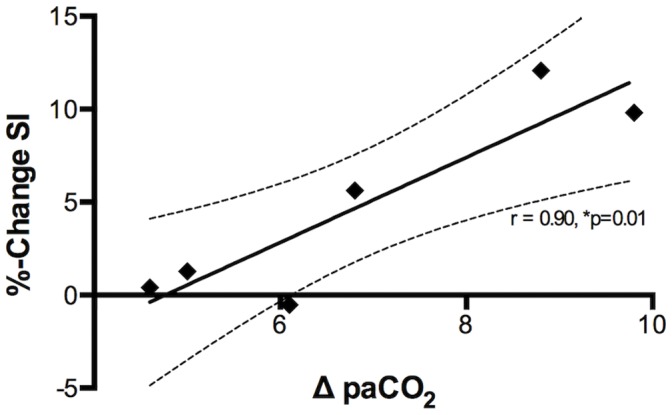
Correlation of myocardial SI in oxygenation-sensitive CMR images to changes in paCO_2_. Differences in paCO_2_ (mmHg) are plotted against the % change in myocardial SI (n = 6). The data shows excellent linear correlation (r = 0.90, *p* = 0.010). The dashed lines represent the 95% confidence interval.

Between 6 and 8 BOLD SSFP cine series were acquired during the apneic periods. During apnea, there was a strong, yet non-significant trend for an increase of myocardial SI (4.8±2.2%; *p* = 0.077; Figure 2). The increase in myocardial SI was linearly correlated with the change in paCO_2_ (r = 0.90, *p* = 0.010; Figure 3), while there was no correlation between myocardial SI changes and changes of paO_2_. SI in the LV blood pool decreased during apnea by 8.0±3.0% (*p* = 0.047). The relative drop in blood pool SI detected by oxygenation-sensitive MR was similar to the 9.9±3.5% (*p* = 0.037) drop in calculated SaO_2_. Heart rate was not correlated with changes in SI.

## Discussion

The data indicate that long breath-holds lead to a consistent, transient decrease of the myocardial deoxyhaemoglobin fraction, which can be detected by oxygenation-sensitive imaging. SI changes in oxygenation-sensitive images have to be evaluated in the context of tissue blood flow, vascular homogeneity, and systemic Hb oxygenation. Our endpoints however refer to repeated measurements and thus are not sensitive to stable systematic confounders. Most importantly, all experiments have been performed under steady conditions with respect to oxygen consumption. The observed changes therefore reflect the tissue response to the increased CO_2_ caused by the breath-hold.

### CMR of Myocardial Oxygen

Under dynamic conditions, various factors determine myocardial tissue oxygenation. While during constant blood flow (and thus constant O_2_ supply), an increased O_2_ demand would result in de-oxygenation and thus a SI decrease, a decreased O_2_ demand would have an opposite effect. In the setting of constant O_2_ demand, an increase of blood flow leads to a decreased fraction of de-oxyHb, exhibiting the BOLD effect used for oxygenation-sensitive CMR imaging.

The observed BOLD-effect is predominantly due to changes of inhomogeneities in the magnetic field occurring in and around the myocardial capillaries [14]. Approximately 90% of the intra-myocardial blood volume and thus haemoglobin resides in these vessels [15]. Under resting conditions only a fraction of the capillaries in the myocardium are believed to be perfused. In the presence of a vasodilator without an increase in myocardial oxygen-consumption in healthy myocardium, the opening of arterioles [16] increases the total oxygen supply without a matching change of the demand and thereby reduces the de-oxyHb content in the capillary beds. Accordingly, this leads to an increase of T2* of the tissue in the affected perfusion bed [6], [17].

Several BOLD-sensitive approaches have been used in experimental settings to assess myocardial oxygenation as a marker for coronary artery disease, often using adenosine or dipyridamole as a vasodilator. While healthy vessels dilate and lead to an increase in myocardial SI [7], myocardium subtended by stenotic vessels may show a blunted response to the vasodilatory trigger or even a decrease in myocardial BOLD-SI as supported by a similar study in an experimental model [18]. Recently, Manka et al. reported shortening of T2* times during adenosine infusion in segments subtended by stenotic coronary arteries at 3T [19].

Importantly, SI changes in BOLD-sensitive images acquired during steady conditions have been shown to exclusively reflect changes of Hb oxygenation [9], [20]. Of note, such changes are linearly correlated with oxygenation of coronary sinus blood. While an ultimate proof of changes of oxygenation on a tissue level would appear helpful, the required methodology, i.e. invasive procedures in the tissue would interfere with the molecular response and thus become a very strong confounder itself. Previous canine studies at 1.5T have found good correlations between BOLD SI increases and the increases of microsphere-measured flow during adenosine indicating that regional changes in BOLD SI were directly related to the relative change in blood flow [18], [21]. This could be reproduced during adenosine mediated vasodilation at 3T in patients with coronary artery disease, where moderate but significant correlations were observed between the BOLD SI change and both, regional myocardial blood flow and coronary flow reserve measurements determined by positron emission tomography with oxygen 15-labeled water [22]. Li et al. compared the SI response to vasodilation with and without increased cardiac workload and could show that SI changes in BOLD CMR images reflect primarily oxygenation and not blood flow [23]. More recently however, Arnold et al. demonstrated that an increase in myocardial blood flow is also correlated to changes in BOLD-SI, until oxygenation reaches a plateau. At this plateau oxygenation is maximal and cannot be increased with further increase of blood flow [24]. Therefore, there is solid evidence that change in blood flow has a secondary effect on SI in oxygenation-sensitive CMR images only, which is simply due to the associated impact on oxygen supply.

### CO_2_ as a Coronary Vasodilator

To respond to changes in myocardial oxygen consumption, coronary blood flow is tightly controlled using several metabolic mechanisms to maintain adequate tissue oxygenation. CO_2_, a major byproduct of all energy-dependent processes, plays a strong role in this regulation [25], tightly coupling demand and supply by its vasodilatory effect on local blood circulation [4], [26]. The vasodilatory effect of CO_2_ and its impact on blood flow [3] and volume [17] have been demonstrated. Changes in systemic paCO_2_ through breathing maneuvers therefore mimic the effect of vasodilators such as adenosine and dipyridamole.

Our observation that there is a trend toward increased myocardial SI despite a parallel SI drop in arterial blood suggests, that the CO_2_-mediated increase in vessel diameter and thus perfusion can fully compensate for the drop in arterial haemoglobin saturation during a 60s breath-hold in swine.

Short breath-holds in humans were sufficient to demonstrate increases in cerebral BOLD-sensitive SI in the brain [27]. However, results in animal models were inconsistent [28]. In a rat model of Kannurpatti et al. a drop in SI intensity was observed during a 20 second breath-hold when the animals were ventilated with room air. Increasing the FiO_2_ to 100% resulted in increased SI in BOLD-sensitive images. Of note, there was a larger increase in cerebral blood flow when these changes were induced at a baseline with room air, corresponding with lower paO_2_ levels. A model of hypercapnia, hypocapnia and hyperoxaemia in anaesthetized dogs, however, showed rather inhomogenous results for different regions of the brain [29]. Through the inhalation of different gas mixtures, BOLD signal intensity in the human brain increased during hypercapnia, yet a larger increase was observed during hypercapnic hyperoxia [30].

In contrast to previous studies, which used rather extreme hypercapnic states to elicit changes in coronary blood flow in invasive studies [31]–[33], we found small increases of paCO_2_ to be sufficient to exhibit increases of SI. The CO_2_-dependent coronary flow increase led to an excess oxygen supply which, given the unchanged oxygen demand, expectedly resulted in a lower de-oxyHb fraction and thus increased SI in the oxygenation-sensitive images.

During long breath-holds, increases in SI attributed to blood flow may be counteracted by a decrease caused by blood Hb desaturation. As expected from those physiological considerations, we observed a decrease of the SI of blood reflecting decreased blood oxygenation during prolonged breath-holds. A drop of arterial blood SI likely blunted the effect of the blood flow increase on myocardial oxygenation. Of note, other studies report an additive effect of hypoxia to hypercapnic increases of myocardial blood flow [31]–[34]. To further understand the competing effects of a breath-hold and the resulting CO_2_ increase on myocardial tissue oxygenation, it would have been interesting to separate both Hb-desaturation and hypercapnia in this pilot study. We were however also interested in applying a model which would be used as a diagnostic approach in patients. Our results may in fact indicate a diagnostic utility for assessing coronary artery function without the need for the systemic or intracoronary application of pharmacologic vasodilators, which often is associated with side effects. Yet, the clinical feasibility and utility of this approach in spontaneously breathing volunteers/patients and the ability of CO_2_ as a direct coronary vasodilator to distinguish diseased from healthy vessels such as adenosine requires further investigation.

We encountered a decrease of blood pressure, which may have confounded our results by decreasing myocardial workload and we cannot exclude that this was in part due to or modified by cardio-suppressive effects of the anaesthesia. Changes in myocardial SI and blood pressure, however, were not correlated. Thus a significant confounding effect is highly unlikely.

Finally, our model may also be limited by the application of general anaesthesia, since several anaesthetic drugs are known to affect the vascular tone. However, the concept of balanced anaesthesia has likely reduced cardiovascular side effects.

### Conclusion

Despite a drop in blood oxygen levels, myocardial oxygenation is maintained throughout long breath-holds and is linearly correlated with the parallel increase of arterial CO_2_, a known coronary vasodilator. Breathing maneuvers in combination with oxygenation-sensitive CMR may be useful as a diagnostic test for coronary artery function.
