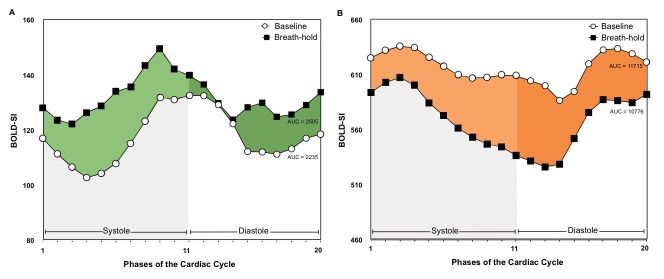# Correction: Impact of Intermittent Apnea on Myocardial Tissue Oxygenation—A Study Using Oxygenation-Sensitive Cardiovascular Magnetic Resonance

**DOI:** 10.1371/annotation/fc3d5cb2-b83c-49ec-a770-a87d17de5ae1

**Published:** 2013-10-10

**Authors:** Dominik P. Guensch, Kady Fischer, Jacqueline A. Flewitt, Matthias G. Friedrich

Figure 1 has been updated for clarity. Please see the corrected Figure 1 here: 

**Figure:**